# Prognostic value of four insulin resistance indices in predicting new-onset hypertension: a retrospective cohort study

**DOI:** 10.3389/fendo.2026.1824452

**Published:** 2026-05-19

**Authors:** Xinyue Yang, Qingwei He, Wenfei Zha, Bowen Li, Qingbo Lv, Yukun Cao, Haitao Zhang

**Affiliations:** 1 Graduate School, Hebei North University, Zhangjiakou, Hebei, China; 2 The Fifth School of Clinical Medicine, Air Force Clinical Medical School, Anhui Medical University, Hefei, Anhui, China; 3 Department of Dermatology, The Second Affiliated Hospital of Anhui Medical University, Hefei, Anhui, China; 4 Department of Cardiology, Air Force Medical Center, Air Force Medical University, People's Liberation Army (PLA), Beijing, China

**Keywords:** hypertension, insulin resistance, risk prediction, metabolic indices, triglyceride-glucose index, cholesterol, high-density lipoprotein, glucose index (CHG index)

## Abstract

**Background:**

Primary prevention of hypertension depends on early detection of high-risk individuals. Several useful risk-stratification tools are provided by lipid-glucose-insulin obtained from standard blood testing. This study compared the predictive performance of four indices for incident hypertension: Atherogenic Index of Plasma (AIP), Triglyceride-Glucose (TyG) index, Cholesterol-HDL-Glucose (CHG) index, and Metabolic Score for Insulin Resistance (METS-IR).

**Methods:**

A total of 1079 adults with initially normal blood pressure were enrolled between January 2016 and January 2019 and followed up until January 2024. Participants were classified according to incident hypertension status during follow-up, the participants were divided into the hypertension group and the non-hypertension group. Multivariable Cox proportional hazards models were used to assess the associations between the four insulin resistance indices and the incidence of hypertension, adjusting for a comprehensive set of clinical and biochemical confounders. Time-dependent receiver operating characteristic (ROC) curve analysis was used to assess predictive accuracy at 12, 36, and 60 months.

**Results:**

Among the participants, 297 developed hypertension during follow-up. In the fully adjusted model, only the CHG index maintained a statistically independent association with hypertension risk. Each unit increase in the CHG index was associated with a more than twofold higher risk of hypertension (Hazard Ratio [HR] 2.186, 95% Confidence Interval [CI] 1.269-3.766). However, the AIP, TyG index, and METS-IR index showed no statistical significance after final adjustment. According to time-dependent ROC analysis, both the CHG index and METS-IR showed excellent and consistent discriminative capacity across initial, medium, and long-term follow-up (AUCs consistently >0.71). At the follow up time of 36 and 60 months, the CHG index exhibited the highest AUC values. The TyG index showed intermediate predictive performance, whereas AIP consistently exhibited the lowest predictive power at all intervals.

**Conclusion:**

The CHG index demonstrated the most independent association with incident hypertension, better than the AIP, TyG, and METS-IR indices in predictive accuracy.

## 1 Introduction

Hypertension is still one of the major worldwide public health concerns. It is a major cause of death and disability from cardiovascular disease (CVD), affecting an estimated 1.4 billion individuals aged 30–79 worldwide (1). The rates of controlling hypertension have not considerably improved despite an increasing awareness of the risks. Over the past thirty years, the total number of people with hypertension has almost doubled, with the largest increase seen in low- and middle-income countries (LMICs) (2, 3). This increasing burden highlights the urgent need for enhanced primary prevention strategies, which hinge on the early identification of at-risk individuals. Effective prevention requires accurate risk classification. However, traditional risk factors such as age and family history often make it difficult to implement individualized interventions (4). Single biomarkers like fasting plasma glucose (FPG) and cholesterol have certain predictive value, but comprehensive indicators can provide a more comprehensive approach (5–7). These indices can greatly enhance risk assessment by more accurately representing pathophysiological processes as insulin resistance (IR) and dyslipidemia (8). IR is a key clinical feature of metabolic syndrome and plays a significant role in the pathogenesis of hypertension (9–11). It raises blood pressure and peripheral vascular resistance by causing dyslipidemia, vascular endothelial dysfunction, and a pro-inflammatory state (9, 12, 13). The Triglyceride-Glucose (TyG) index has emerged as a simple, reliable, and low-cost surrogate marker of IR, demonstrating a significant association with incident hypertension in various populations (10, 14). Similarly, the Atherogenic Index of Plasma (AIP) reflects atherogenic dyslipidemia and IR, and has been linked to cardiovascular risk and hypertension (12). Recently, two novel indices have been proposed. Compared to the TyG index, the cholesterol, high-density lipoprotein (HDL), and glucose index (CHG) has demonstrated better diagnostic performance for type 2 diabetes mellitus (T2DM) (15), but their association with hypertension risk remains unclear. A non-insulin-based measure of IR, the Metabolic Score for Insulin Resistance (METS-IR) has been positively associated with hypertension in cross-sectional studies (16, 17). The AIP and TyG indices are useful tools for assessing IR and cardiovascular risk, but they primarily focus on triglyceride and glucose levels. In contrast, the CHG index includes total cholesterol (TC), which is a key factor in atherosclerosis. And the METS-IR integrates body mass index (BMI), a direct measure of adiposity, which is a central driver of hypertension. It remains unclear whether these more comprehensive indices offer superior predictive value for incident hypertension compared to the traditional AIP and TyG. This study directly compared all four indices to find the strongest predictor of new-onset hypertension.

## 2 Materials and methods

### 2.1 Study design and population

This was a retrospective cohort study conducted at the Air Force Medical Center. Participants who underwent a routine health examination between January 2016 and January 2019 were identified from the hospital’s electronic medical record system. The baseline was established as the date of the initial health assessment (2016-2019). Follow-up data, including blood pressure measurements and medication records, were extracted from the hospital’s electronic medical record system until January 2024. We included participants who met the following criteria at baseline: 1) age ≥ 18 years; 2) normotensive at baseline, defined retrospectively as having systolic blood pressure (SBP) < 140 mmHg and diastolic blood pressure (DBP) < 90 mmHg according to the 2024 ESC guideline criteria, with no self-reported history of hypertension and no use of antihypertensive medications (18); and 3) complete clinical data.

**Figure 1:**
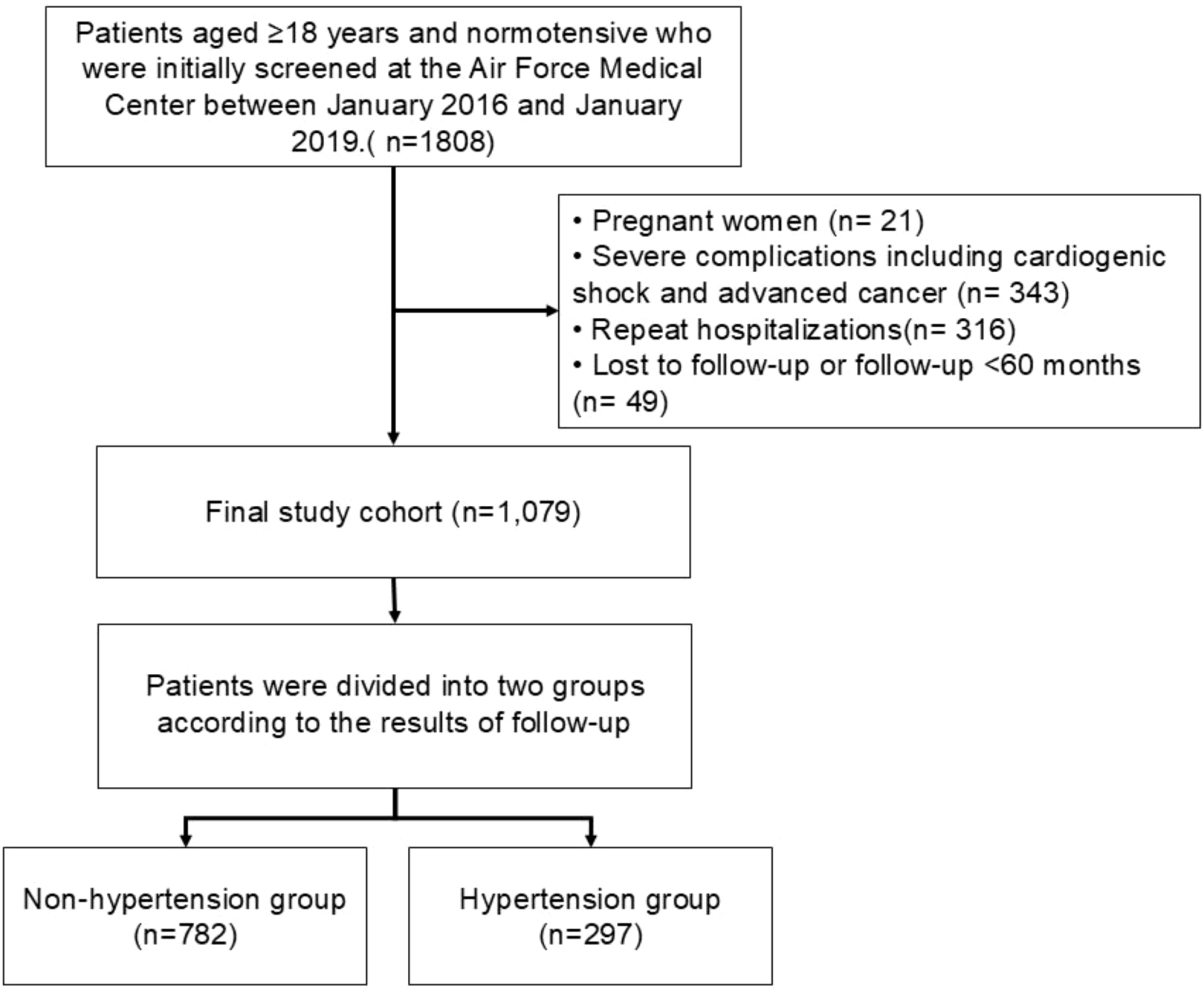
Flowchart of participant selection in the study.

Following the exclusion of pregnant women, individuals with repeated hospital admissions, those with significant missing key data, failure or loss to follow-up, and patients suffering from severe complications such as cardiogenic shock or advanced cancer, a total of 1079 participants were ultimately included in the cohort. Among the 1079 participants, there were 782 individuals with normotension and 297 individuals with hypertension. The average age of the overall cohort was 30.76 ± 7.284 years. **Figure 1** presents a flowchart detailing the criteria for participant inclusion and exclusion.

This study complies with the principles of the Declaration of Helsinki and was approved by the Ethics Committee of the Air Force Medical Center of the Chinese People’s Liberation Army (2024-67-PJ01). Given the retrospective design and the complete de-identification of all patient data, the Institutional Review Board of the Air Force Medical Center granted a waiver of informed consent.

### 2.2 Data collection and study variables

Patients’ clinical information, including basic clinical characteristics, behavioral characteristics, laboratory parameters, past medical history, and IR indices were collected at admission from the Hospital Information System. Basic clinical characteristics encompassed age, gender, height, weight, BMI, pulse, SBP, and DBP. Behavioral characteristics comprised smoking status, smoking duration, smoking intensity, drinking status, family history, and marital status. Laboratory parameters included key metrics such as the lipid profile (LDL-C, HDL-C, TC, TG), measures of renal function and metabolism (BUN, FPG, blood creatinine, uric acid), blood routine and biochemical indices (ALB, RBC, Hb, MCH, MCV, MCHC), and inflammatory cell counts (monocyte, lymphocyte, neutrophil). All laboratory parameters were measured from fasting overnight (>8 hours) peripheral venous blood samples. Past medical history consisted of documented histories of atherosclerosis, hepatic steatosis, hyperlipidemia, hyperuricemia, renal disease, and impaired glucose tolerance (IGT). IR indices (AIP, CHG, TyG, and METS-IR) were calculated using their respective established formulas.

### 2.3 IR indices

The IR indices evaluated in this study included the Atherogenic Index of Plasma (AIP), Triglyceride-Glucose (TyG) Index, cholesterol, HDL, glucose (CHG) Index, and Metabolic Score for Insulin Resistance (METS-IR) Index. These indices were calculated using the following established formulas:

1. AIP = Lg [TG (mmol/L)/HDL-C (mmol/L)] (13)
2. TyG = Ln [TG (mg/dL) * FPG (mg/dL)/2] (10)
3. CHG = Ln [TC (mg/dL)*FPG (mg/dL)/2*HDL (mg/dL)] (15)
4. METS-IR = (Ln [(2 * FPG (mg/dL)) + TG (mg/dL)] * BMI (kg/m^2^))/(Ln [HDL-C (mg/dL)] (16)

### 2.4 Endpoint and follow-up

The primary outcome of this study was incident hypertension, defined as the first occurrence of either a SBP ≥ 140 mmHg, a DBP ≥ 90 mmHg, or the initiation of antihypertensive drug therapy during the follow-up period, with the diagnosis confirmed by at least two separate measurements. Trained investigators ascertained the survival status and collected outcome data through a combination of reviewing subsequent inpatient and outpatient electronic medical records and conducting structured telephone interviews. The follow-up period commenced on the date of the baseline admission and extended until the diagnosis of hypertension, patient death, or the end of the follow-up period, whichever occurred first.

### 2.5 Statistical analysis

Statistical analyzes were performed using R software (version 4.4.1) and SPSS (version 27.0). Continuous variables with normal distribution were expressed as mean ± standard deviation and compared between groups using an independent samples t-test. Non-normally distributed variables were presented as median (interquartile range) and compared using the Mann-Whitney U test. Categorical variables were expressed as frequencies (percentages) and compared using the Chi-square test. Three multivariable Cox proportional hazards models were used to assess the relationships between IR indices (AIP, CHG index, TyG index, and METS-IR) and incident hypertension. Model 1 was unadjusted. Model 2 was adjusted for age, gender, pulse, SBP, DBP, smoking, drinking, and family history. Model 3 was further adjusted for LDL-C, uric acid, ALB, platelet count, monocyte count, neutrophil count, atherosclerosis, hepatic steatosis, hyperlipidemia, hyperuricemia, renal disease, and IGT. Multicollinearity among the four IR indices and other covariates was assessed by calculating the variance inflation factor (VIF) for each variable in the fully adjusted model (Model 3). The results were expressed as hazard ratios (HRs) with 95% confidence intervals (CIs). The predictive performance of the four indices for hypertension risk was assessed using time-dependent receiver operating characteristic (ROC) curves at 12, 36, and 60 months, with area under the curve (AUC) values calculated for comparison. The cumulative incidence of hypertension among tertiles of each IR index was visualized using Kaplan-Meier curves and compared with the log-rank test. Restricted cubic spline (RCS) analysis was employed to explore potential nonlinear relationships between continuous indices and hypertension risk. A two-sided P < 0.05 was considered statistically significant.

## 3 Results

### 3.1 Baseline characteristics

Baseline characteristics of the 1079 participants, stratified by hypertension status during follow-up, are presented in **Table 1**. The average age of the study population was 30.76 ± 7.284 years, including 541 male patients (50.1%). Participants who developed hypertension (n=297) were significantly older and had higher BMI, systolic and diastolic blood pressure, and neutrophil counts compared to those who remained normotensive (n=782). Additionally, the hypertension group had a higher proportion of smokers, longer smoking history, and a greater prevalence of family history of hypertension (all *P* < 0.05), but a lower proportion of drinkers (*P* = 0.008). Metabolically, the hypertension group exhibited a more adverse profile, including elevated LDL-C, total cholesterol, triglycerides, fasting blood glucose, and uric acid, as well as lower HDL-C levels (all *P* < 0.001, **Table 1**). Furthermore, all four IR indices were significantly higher in the hypertension group compared to the non-hypertension group (all *P* < 0.001), including the AIP, CHG index, TyG index, and METS-IR. No significant intergroup differences were observed in other variables (*P* > 0.05).

**Table 1:** Baseline characteristics of the non-hypertension and hypertension groups.

Variables	Total (n =1079)	Non-hypertension group (n =782)	Hypertension group (n =297)	t/Z/X^2^	P value
Basic Clinical Characteristics					
Age (years)	30.76 ± 7.284	28.72 ± 5.668	36.14 ± 8.267	14.257	<0.001
Gender, male (%)	541(50.1)	386(49.4)	155(51.9)	0.534	0.465
Height (cm)	173.50(171.00, 176.00)	173.00(171.00, 176.00)	174.00(171.00, 177.00)	-1.347	0.178
Weight (kg)	72.00(67.00, 75.00)	70.00(65.00, 74.00)	74.00(71.00, 80.00)	-9.294	<0.001
BMI (kg/m^2^)	23.62(22.23, 24.86)	23.24(22.13, 24.39)	24.62(23.53, 25.93)	-10.341	<0.001
Pulse (beats/min)	69.00(64.00, 74.00)	69.00(62.00, 74.00)	69.00(69.00, 73.00)	-3.971	<0.001
SBP (mmHg)	113.00(110.00, 122.00)	112.00(109.00, 114.00)	128.00(123.00, 133.00)	-22.125	<0.001
DBP (mmHg)	74.00(68.00, 81.00)	71.00(66.00, 75.00)	82.00(80.00, 85.00)	-21.187	<0.001
Behavioral Characteristics					
Smoking, n (%)	444(41.1)	297(38.0)	147(49.5)	11.787	<0.001
Smoking Duration (years)	0.00(0.00, 10.00)	0.00(0.00, 10.00)	0.00(0.00, 14.00)	-4.321	<0.001
Smoking Intensity (n/day)	0.00(0.00, 8.00)	0.00(0.00, 5.00)	0.00(0.00, 10.00)	-4.459	<0.001
Drinking, n (%)	916(85.1)	679(86.8)	237(80.3)	7.095	0.008
Family History, n (%)	68(6.3)	24(3.1)	44(14.8)	50.291	<0.001
Marital Status (married %)	985(91.3)	722(92.3)	263(88.6)	3.857	0.050
Laboratory Parameters					
LDL-C(mmol/L)	2.59 ± 0.651	2.50 ± 0.595	2.81 ± 0.736	6.582	<0.001
HDL-C(mmol/L)	1.26 ± 0.275	1.29 ± 0.271	1.20 ± 0.276	-4.773	<0.001
TC(mmol/L)	4.37 ± 0.824	4.26 ± 0.740	4.69 ± 0.944	7.079	<0.001
TG(mmol/L)	1.05(0.77, 1.49)	0.97(0.73, 1.34)	1.33(0.91, 2.00)	-8.182	<0.001
BUN(mmol/L)	5.50(4.70, 6.30)	5.40(4.70, 6.30)	5.50(4.80, 6.20)	-0.382	0.702
FPG (mmol/L)	4.80(4.50, 5.20)	4.70(4.50, 5.10)	5.10(4.78, 5.50)	-10.254	<0.001
Blood Creatinine (μmol/L)	80.00(72.00, 88.00)	80.00(72.00, 88.00)	80.00(72.00, 87.00)	-0.293	0.770
Uric Acid (μmol/L)	356.86 ± 75.098	343.95 ± 69.373	390.79 ± 79.015	8.985	<0.001
ALB (g/L)	45.46 ± 2.782	45.76 ± 2.716	44.67 ± 2.805	-5.805	<0.001
RBC (×10^9^/L)	4.86 ± 0.409	4.87 ± 0.402	4.85 ± 0.426	-0.507	0.612
MCH (pg)	30.55 ± 1.552	30.53 ± 1.544	30.59 ± 1.575	0.535	0.593
MCV (fL)	90.50 ± 3.795	90.37 ± 3.757	90.85 ± 3.872	1.844	0.065
MCHC (g/L)	337.00(330.00, 344.00)	337.00(330.00, 345.00)	337.00(330.00, 343.00)	-1.001	0.317
Hb (g/L)	148.00(141.00, 155.00)	148.00(141.00, 156.00)	147.00(142.00, 155.00)	-0.250	0.803
Monocyte Count (×10^9^/L)	0.40(0.31, 0.50)	0.40(0.30, 0.50)	0.40(0.37, 0.52)	-2.779	0.005
Lymphocyte Count (×10^9^/L)	2.10(1.76, 2.40)	2.10(1.77, 2.40)	2.10(1.73, 2.40)	-0.389	0.697
Neutrophil Count (×10^9^/L)	3.10(2.60, 3.70)	3.00(2.50, 3.62)	3.20(2.74, 4.00)	-3.579	<0.001
Past medical history, n (%)					
Atherosclerosis	50(4.6)	5(0.6)	45(15.2)	102.580	<0.001
Hepatic Steatosis	66(6.1)	26(3.3)	40(13.5)	38.564	<0.001
Hyperlipidemia	94(8.7)	32(4.1)	62(20.9)	76.239	<0.001
Hyperuricemia	72(6.7)	30(3.8)	42(14.1)	36.705	<0.001
Renal Disease	265(24.6)	177(22.6)	88(29.6)	5.685	0.017
IGT	48(4.4)	21(2.7)	27(9.1)	20.777	<0.001
Insulin Resistance indices					
AIP	-0.076(-0.248, 0.117)	-0.127(-0.272, 0.062)	0.066(-0.145, 0.237)	-8.534	<0.001
CHG Index	5.021 ± 0.294	4.951 ± 0.262	5.206 ± 0.295	13.093	<0.001
TyG Index	8.289(7.980, 8.694)	8.202(7.911, 8.557)	8.592(8.215, 9.063)	-10.009	<0.001
METS-IR	34.057(31.628, 37.224)	33.128(31.004, 35.891)	37.181(34.190, 40.566)	-12.165	<0.001

Values are given as median and interquartile range or numbers and percentages.

BMI, body mass index; SBP, systolic blood pressure; DBP, diastolic blood pressure; TG, triglycerides; TC, total cholesterol; LDL-C, low-density lipoprotein cholesterol; HDL-C, high-density lipoprotein cholesterol; ALB, serum albumin; BUN, Blood Urea Nitrogen; FPG, fasting plasma glucose; RBC, red blood cell count; MCH, mean corpuscular hemoglobin; MCV, mean corpuscular volume; MCHC, mean corpuscular hemoglobin concentration; Hb, Hemoglobin; IGT, impaired glucose tolerance; AIP, atherogenic index of plasma; TyG Index, triglyceride-glucose index; METS-IR, metabolic score for insulin resistance; CHG Index, cholesterol, high density lipoprotein, and glucose index.

### 3.2 Comparison of hypertension status among tertile groups

**Figure 2:**
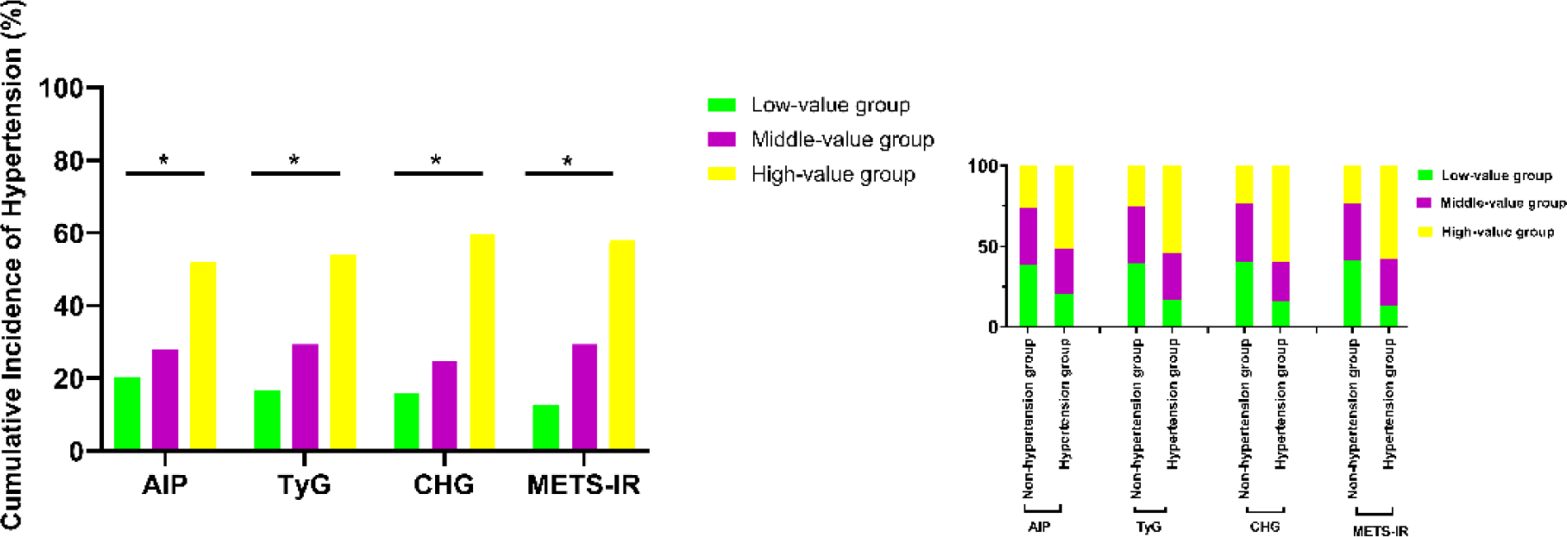
Distribution of hypertension cases by tertiles of insulin resistance indices.

During the follow-up period, participants were stratified into tertiles based on each IR index: the low-value group, the middle-value group, and the high-value group. As shown in **Figure 2**, the cumulative incidence of hypertension varied markedly across these subgroups for all four indices. The high-value groups of AIP, CHG, TyG, and METS-IR consistently exhibited the highest proportions of hypertension cases. Conversely, the low-value groups comprised a notably higher proportion of participants who remained free of hypertension.

### 3.3 Survival analysis and dose-response relationships

**Figure 3:**
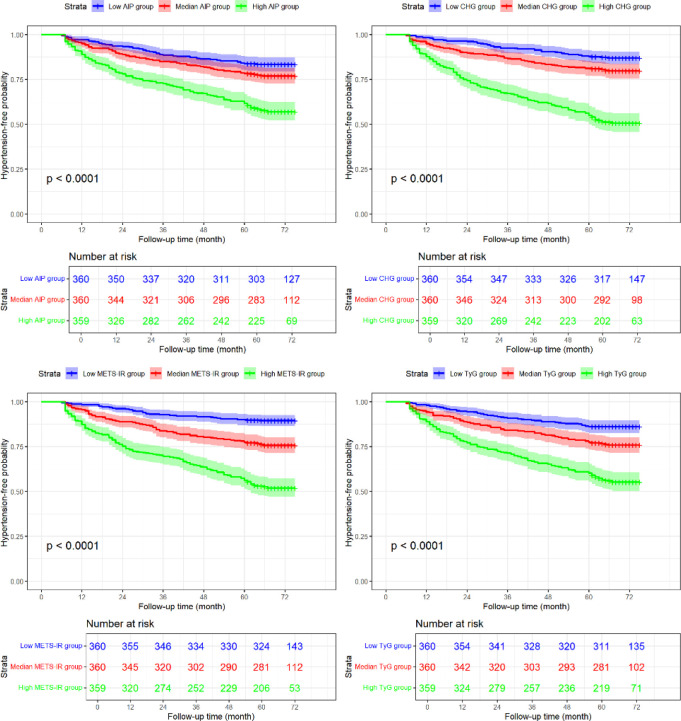
Kaplan-Meier curves showing hypertension-free survival probability according to tertiles of the four insulin resistance indices.

Kaplan-Meier curves for hypertension-free survival, stratified by tertiles of each metabolic index, are shown in **Figure 3**. The cumulative incidence of hypertension was significantly higher in the highest tertile compared to the lowest tertile for all four indices (log-rank *P* < 0.0001 for all). The survival curves for the highest tertiles of the CHG index and METS-IR declined more rapidly.

### 3.4 Associations between IR indices and hypertension risk

Prior to the main Cox regression analysis, multicollinearity among the variables in the fully adjusted model (Model 3) was evaluated. All VIF values were well below the conventional threshold of 5. The associations between the IR indices and incident hypertension risk were assessed using Cox proportional hazards models, with results detailed in **Table 2**.

**Table 2:** Cox regression models for the association between AIP, CHG index, TyG index, METS-IR, and hypertension risk.

Insulin resistance indices	Model 1		Model 2		Model 3	
	HR (95% CI)	P	HR (95% CI)	P	HR (95% CI)	P
AIP	4.616(3.277, 6.501)	<0.001	1.352(0.890, 2.055)	0.158	1.175(0.729, 1.892)	0.508
AIP<-0.18	Ref		Ref		Ref	
-0.18≤AIP<0.05	1.441(1.034, 2.008)	0.031	0.903(0.640, 1.274)	0.562	0.879(0.618, 1.250)	0.473
AIP≥0.05	3.055(2.266, 4.117)	<0.001	1.342(0.968, 1.862)	0.078	1.201(0.842, 1.712)	0.312
CHG Index	10.787(7.571, 15.369)	<0.001	2.250(1.527, 3.316)	<0.001	2.186(1.269, 3.766)	0.005
CHG<4.87	Ref		Ref		Ref	
4.87≤CHG<5.13	1.648(1.142, 2.378)	0.008	1.231(0.847, 1.788)	0.276	1.228(0.841, 1.793)	0.287
CHG≥5.13	4.880(3.536, 6.734)	<0.001	1.924(1.361, 2.720)	<0.001	1.712(1.153, 2.542)	0.008
TyG Index	2.643(2.219, 3.148)	<0.001	1.190(0.960, 1.475)	0.112	1.157(0.901, 1.486)	0.252
TyG ≤ 8.10	Ref		Ref		Ref	
8.10≤TyG<8.54	1.856(1.311, 2.628)	<0.001	1.280(0.891, 1.840)	0.182	1.193(0.823, 1.729)	0.351
TyG≥8.54	3.933(2.862, 5.405)	<0.001	1.493(1.052, 2.120)	0.025	1.389(0.957, 2.016)	0.084
METS-IR	1.126(1.106, 1.146)	<0.001	1.033(1.008, 1.059)	0.010	1.024(0.997, 1.052)	0.082
METS-IR<32.44	Ref		Ref		Ref	
32.44≤METS-IR<36.06	2.487(1.699, 3.641)	<0.001	1.226(0.828, 1.815)	0.308	1.257(0.821, 1.925)	0.292
METS-IR≥36.06	5.812(4.089, 8.262)	<0.001	1.672(1.151, 2.429)	0.007	1.507(0.872, 2.606)	0.142

Model 1 was unadjusted. Model 2 was adjusted for age, gender, pulse, SBP, DBP, smoking, drinking, and family history. Model 3 was adjusted for Model 2 plus LDL-C, uric acid, ALB, platelet count, monocyte count, neutrophil count, atherosclerosis, hepatic Steatosis, hyperlipidemia, hyperuricemia, renal disease and IGT.

SBP, systolic blood pressure; DBP, diastolic blood pressure; LDL-C, low-density lipoprotein cholesterol; ALB, serum albumin; IGT, impaired glucose tolerance; AIP, atherogenic index of plasma; TyG Index, triglyceride-glucose index; METS-IR, metabolic score for insulin resistance; CHG Index, cholesterol, high density lipoprotein, and glucose index;HR, hazard ratio; CI, confidence interval.

In unadjusted analyzes (Model 1), all four indices were strongly associated with hypertension risk (all *P* < 0.001, **Table 2**). After adjusting for age, gender, pulse, SBP, DBP, smoking, drinking, and family history in Model 2, the strength of the associations was attenuated. In this model, the CHG index (HR 2.250, 95% CI 1.527–3.316, *P* < 0.001), the highest tertile of the TyG index (HR 1.493, 95% CI 1.052–2.120, *P* = 0.025), and the highest tertile of METS-IR (HR 1.672, 95% CI 1.151–2.429, *P* = 0.007) remained significantly associated with increased risk.

After further adjustment for clinical and biochemical confounders (LDL-C, uric acid, ALB, platelet count, inflammatory cells, and comorbidities) in the fully adjusted Model 3, only the CHG index maintained a statistically independent association with hypertension risk, both as a continuous variable and when categorized in its highest tertile. Each unit increase in the CHG index was associated with a more than twofold higher risk of developing hypertension (HR 2.186, 95% CI 1.269–3.766, *P* = 0.005). When analyzed by tertiles, participants in the highest CHG tertile had a 71% increased risk (HR 1.712, 95% CI 1.153–2.542, *P* = 0.008) compared to those in the lowest tertile. In contrast, the associations for AIP, TyG index, and METS-IR were attenuated and lost statistical significance in the fully adjusted model.

**Figure 4:**
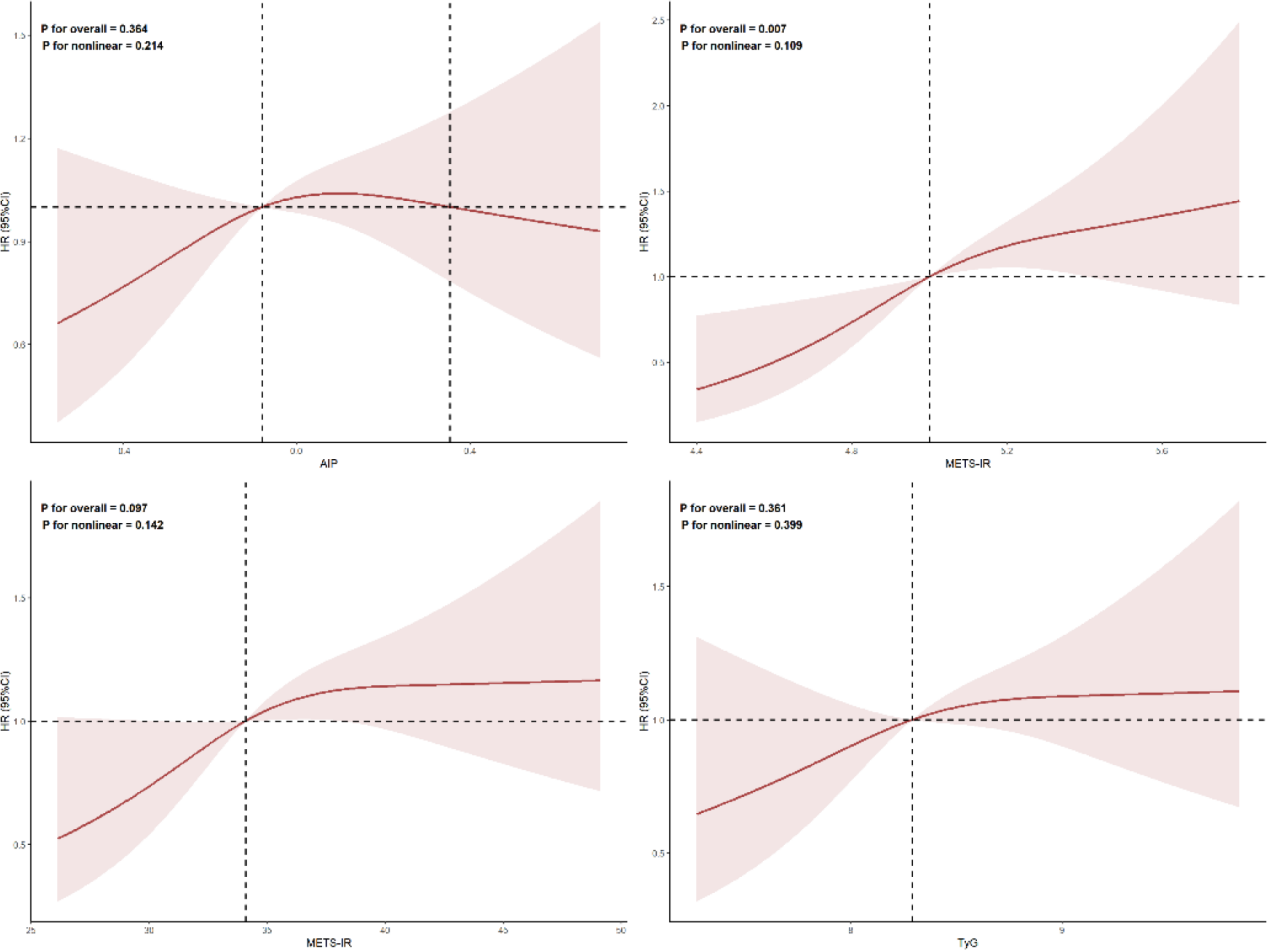
RCS analysis of the associations between IR indices and hypertension risk.

To further characterize the exposure-response relationship, RCS analysis was performed within the fully adjusted Cox model (Model 3, **Figure 4**). A significant linear association was observed between the continuous CHG index and the log hazard of hypertension (*P* for overall = 0.007, *P* for nonlinear = 0.109), indicating a steadily increasing risk of hypertension with higher CHG values. In contrast, the dose-response curves for the continuous AIP (*P* for overall = 0.364), TyG index (*P* for overall = 0.361), and METS-IR (*P* for overall = 0.097) were not statistically significant in the fully adjusted model.

### 3.5 Predictive performance comparison

**Figure 5:**
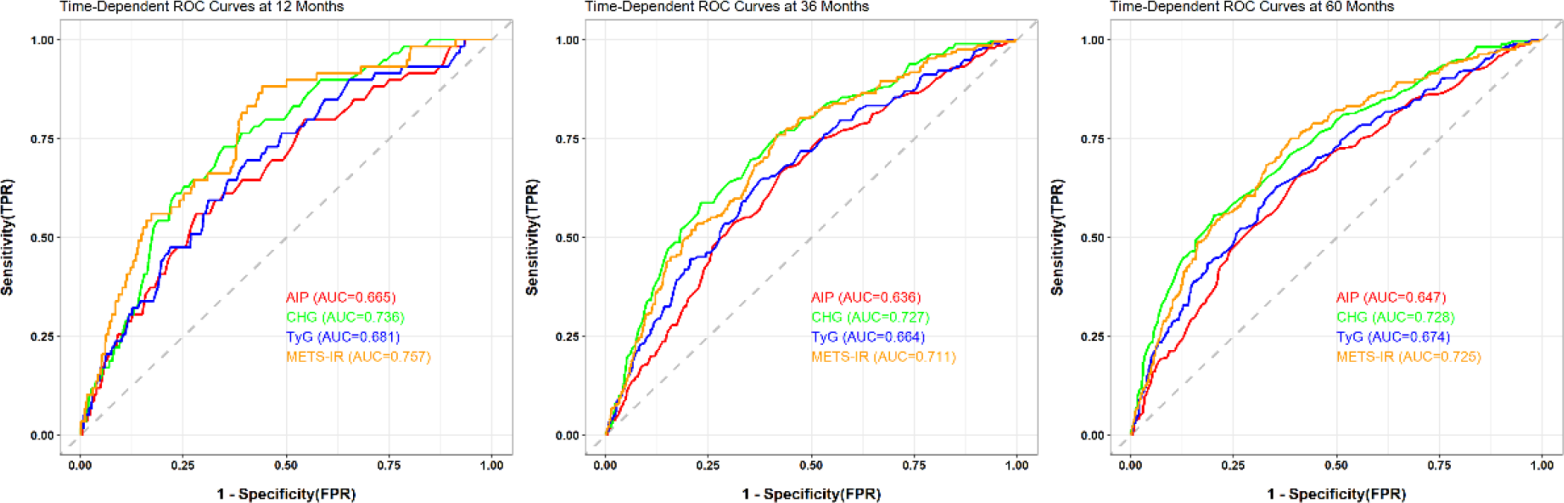
Time-dependent ROC, receiver operating characteristic curves for predicting incident hypertension by the four metabolic indices.

The time-dependent discriminative ability of each index was compared using ROC curve analysis (**Figure 5**). At 12 months, the METS−IR showed the highest discriminative ability with an AUC of 0.757, followed closely by the CHG index (AUC = 0.736). The TyG index exhibited an intermediate AUC of 0.681, while the AIP demonstrated the lowest predictive accuracy (AUC = 0.665). At 36 months, the CHG index achieved the highest predictive accuracy with an AUC of 0.727, followed by the METS−IR (AUC = 0.711). The TyG index yielded an AUC of 0.664, while the AIP again demonstrated the lowest discriminative power (AUC = 0.636). At 60 months, the CHG index attained the highest AUC (0.728), with METS−IR showing comparable performance (AUC = 0.725). The TyG index displayed an AUC of 0.674, and AIP again presented the lowest value (AUC = 0.647). Throughout the follow−up, the CHG index and METS−IR consistently demonstrated superior and stable predictive performance, with both indices maintaining AUCs above 0.71 at all-time points. In contrast, the TyG index showed intermediate discriminative ability, while AIP consistently exhibited the lowest predictive power across all intervals.

## 4 Discussion

This retrospective cohort study was conducted to directly compare the predictive performance of four IR indices for new−onset hypertension. We used multivariable Cox proportional hazards regression. Three sequentially adjusted models were constructed. The CHG index was identified as the most robust and independent predictor. In the fully adjusted model (Model 3), each unit increase in the CHG index was associated with a more than twofold higher risk of hypertension (HR = 2.186, 95% CI: 1.269–3.766, *P* = 0.005), and participants in the highest tertile of the CHG index had a 71% higher risk (HR = 1.712, 95% CI: 1.153–2.542, *P* = 0.008) compared to the lowest tertile. Time−dependent ROC analysis showed that the discriminative ability of the CHG index was robust and stable throughout time. The METS−IR exhibited a slightly higher AUC at 12 months (AUC = 0.757), and the CHG index achieved the highest AUC values at both 36 and 60 months (0.727 at 36 months, and 0.728 at 60 months). The superior performance of the CHG index highlights its potential as a practical tool for early risk stratification, which is crucial for addressing the growing public health burden of hypertension.

IR is a core pathophysiological mechanism implicated in the development of hypertension (19, 20), potentially promoting vascular dysfunction through oxidative stress, chronic inflammation, and sympathetic overactivation (21, 22). Evidence shows that the incidence of T2DM in patients with hypertension is approximately 2.5 times that in patients with normal blood pressure, and hypertension often occurs before diabetes (22). This interplay highlights IR as a key driver of cardiometabolic comorbidity. Given the pivotal role of IR in hypertension, practical and accessible surrogate markers derived from routine laboratory parameters are essential for early risk stratification. Several IR-related indices, including AIP, TyG index, CHG index, and METS-IR, have therefore been proposed as feasible tools for assessing metabolic risk and predicting hypertension.

Among the four IR indices studied, only the CHG index showed a strong and independent link to new-onset hypertension after accounting for lipid levels, inflammatory markers, and other health conditions. This highlights the specific value of the CHG index in predicting hypertension risk, beyond its connection to general metabolic problems. The index also exhibited the best overall predictive performance in ROC analysis, maintaining the highest or near-highest AUC values throughout follow-up. This provides support for its use in long-term risk assessment. The strength of the CHG index probably comes from its ability to capture several related pathways involved in hypertensive vascular disease. The TyG index mainly reflects triglyceride and glucose metabolism related to IR (23). The AIP focuses on atherogenic lipids. In contrast (24), the CHG index uniquely combines TC, HDL-C, and FPG. This combination captures three key pathophysiological processes: atherogenic burden from high TC, reduced vascular protection from low HDL-C, and endothelial dysfunction driven by high blood glucose. High TC promotes atherosclerotic plaque. Low HDL-C is linked to impaired cholesterol clearance and weaker anti-inflammatory and antioxidant activity (25). At the same time, high blood glucose causes oxidative stress, endothelial damage, and vascular remodeling (26–28). By quantifying this confluence of dyslipidemia and dysglycemia, the CHG index effectively represents the aggregate risk that potently accelerates the vascular dysfunction underlying hypertension (15, 29, 30). Our findings align with evidence supporting the CHG index’s predictive capacity for other cardiometabolic endpoints, including cardiovascular disease and type 2 diabetes (15, 31). However, other research presents a more complex picture. Some studies report a U-shaped or J-shaped relationship between the CHG index and mortality in people with advanced metabolic disease, meaning both very low and very high values may indicate a higher risk (32, 33). Also, while the CHG index often has high specificity, its sensitivity can be moderate. Its performance is sometimes similar to the TyG index, especially in ethnically diverse groups (34). These differences may come from variations in study populations, existing health conditions, lifestyle factors, and genetic backgrounds. Our research has extended the use of the CHG index to new-onset hypertension. However, more mechanistic and prospective studies are needed to clarify its exact role in hypertension development and to confirm its usefulness across different populations and clinical settings.

The METS-IR index, which integrates FPG, TG, HDL-C, and BMI, is designed to provide a comprehensive assessment of metabolic health by capturing dysglycemia, dyslipidemia, and adiposity (16). Its incorporation of BMI, a proxy for visceral adiposity, allows it to reflect a key driver of IR and systemic inflammation (16, 35, 36). This explains its established correlation with ectopic fat and predictive value for cardiometabolic diseases (37). In our unadjusted analysis, METS−IR showed a strong association with hypertension risk, consistent with studies that have linked higher METS−IR levels to incident hypertension and arterial stiffness (35, 38). However, its strong performance in earlier models and subsequent attenuation after full adjustment (Model 3) suggest that its predictive signal for hypertension is largely mediated through pathways related to adiposity and its associated metabolic disturbances, which were accounted for in the final model (17, 35). Similar attenuation of METS−IR’s independent association after comprehensive adjustment has been noted in other cohorts (39). Some studies report that METS−IR remains an independent predictor of hypertension, particularly in non−diabetic (40). These discrepancies may stem from differences in population characteristics, adjustment variables, or the relative contribution of visceral adiposity to the metabolic profile. Our findings align with the view that METS−IR is a valuable indicator of general metabolic health, while its specific link to incident hypertension in this population appears to be mediated predominantly by obesity−related pathways.

In contrast, the TyG index, a validated surrogate for IR (41, 42), showed a strong initial association with hypertension risk in our cohort, consistent with the known mechanisms linking hyperinsulinemia, sympathetic overactivation, and impaired vasodilation to blood pressure elevation (43, 44). Furthermore, a meta-analysis has confirmed the positive correlation between the TyG index and hypertension risk across diverse populations (45). Nevertheless, its predictive independence was not sustained after comprehensive adjustment in our analysis. This pattern resembles findings from studies on young adults, where several IR indices lost predictive significance for overall hypertension after full adjustment, with the TyG index retaining association only with specific subtypes, such as stage II combined hypertension and isolated diastolic hypertension (46). Similarly, the AIP reflects the balance between pro-atherogenic and anti-atherogenic lipid particles (47). Elevated AIP may increase cardiovascular risk in hypertensive individuals by promoting a pro-oxidant state and endothelial dysfunction through atherosclerosis (48). Prior research has demonstrated AIP’s predictive value for cardiovascular events and its close link with glucose metabolic abnormalities (13, 49), which often coexist with hypertension (50). However, its predictive power was attenuated in our fully adjusted model. The loss of statistical significance for the TyG index and AIP in the final model suggests that their initial robust association with hypertension risk may be largely mediated through broader elements of the metabolic syndrome. These underlying components are comprehensively captured within the integrative structure of the CHG index.

We used three Cox models with progressively more comprehensive covariate sets to assess the stability of our findings. The CHG index maintained a strong and independent association with incident hypertension after full adjustment in Model 3. In contrast, the associations of the AIP, TyG, and METS-IR indices were highly model dependent. Their strong effects in Model 1 progressively attenuated and lost statistical significance after adjusting for confounders in Model 3. This pattern suggests that the predictive signal of AIP, TyG, and METS-IR is largely explained by their overlap with other measured risk factors. The CHG index may capture a more direct pathway linking IR, dyslipidemia, and hyperglycemia to hypertension development. It may reflect pathways more directly related to hypertension development rather than acting solely as a surrogate marker. As noted by Buse et al., a biomarker can be a significant mediator in unadjusted models but may lose its effect after controlling for variables on the causal pathway or strong confounders (51). Our study cannot definitively establish causality or perfectly separate mediation from confounding. Nevertheless, the differential robustness of the CHG index across adjustment models positions it as a more promising candidate for an independent risk factor. It may reflect an integrated metabolic signal that is causally relevant to hypertension pathogenesis.

The CHG index offer simple, cost-effective, and non-invasive means for early identification of individuals at high risk for hypertension. Calculated from routine laboratory tests and basic anthropometrics, they can be easily integrated into clinical practice and health screenings. Utilizing these indices for risk stratification could enable targeted, early lifestyle interventions in pre-hypertensive stages, potentially delaying or preventing disease onset and reducing the substantial public health burden of hypertension.

## 5 Limitations

Despite its strengths, this study has several limitations. First, its single-center, retrospective design may introduce selection bias and unmeasured residual confounding. Although we adjusted for numerous confounders, residual confounding from unmeasured factors such as dietary habits and physical activity cannot be entirely ruled out. Second, our study population was recruited from a single center in China, which may limit the generalizability of the findings to other ethnicities and healthcare settings. Third, while we used established formulas, the optimal cut-off values for CHG and METS-IR in hypertension prediction require further validation in external cohorts. Furthermore, all IR indices were calculated from single baseline measurements. Their potential fluctuation over time was not assessed, which might affect the accuracy of long-term risk prediction.

Future research should focus on: (1) externally validating the predictive performance and determining optimal cut-off values for the CHG index in prospective, multi-ethnic cohorts; (2) investigating whether changes in the CHG index over time, in response to lifestyle or therapeutic interventions, are associated with corresponding changes in hypertension risk, which would strengthen its utility as a dynamic monitoring tool; and (3) exploring the biological pathways linking the components of the CHG index to hypertension development through mechanistic studies.

## 6 Conclusion

Among the four IR indices evaluated, the CHG index demonstrated the strongest and most independent association with incident hypertension, along with superior predictive performance. Compared with the AIP and TyG index, both the CHG index and METS-IR showed higher predictive efficacy for long-term hypertension risk, as evidenced by their consistent performance across multiple follow-up time points in time-dependent ROC analysis. These composite indices, by effectively integrating key components of metabolic syndrome, provide a valuable tool for early risk stratification.

## Data Availability

The raw data supporting the conclusions of this article will be made available by the authors, without undue reservation.

## Glossary

CVD: cardiovascular disease
AIP: atherogenic index of plasma
ALB: serum albumin
AUC: area under the curve
BMI: body mass index
BUN: blood urea nitrogen
CHG: cholesterol HDL glucose index
DBP: diastolic blood pressure
SBP: systolic blood pressure
FPG: fasting plasma glucose
Hb: hemoglobin
HDL-C: high-density lipoprotein cholesterol
HR: hazard ratio
VIF: variance inflation factor
IGT: impaired glucose tolerance
IR: insulin resistance
LDL-C: low-density lipoprotein cholesterol
METS-IR: metabolic score for insulin resistance
RCS: restricted cubic spline
ROC: receiver operating characteristic
TC: total cholesterol
TG: triglycerides
TyG: triglyceride-glucose index
T2DM: type 2 diabetes mellitus
